# Clinical significance of *p16^INK4A^* and *p14^ARF^* promoter methylation in renal cell carcinoma: a meta-analysis

**DOI:** 10.18632/oncotarget.18826

**Published:** 2017-06-28

**Authors:** Yu Ren, Li Xiao, Guobin Weng, Bingyi Shi

**Affiliations:** ^1^ Department of Urologic Surgery, Ningbo Urology and Nephrology Hospital, Ningbo 315000, People's Republic of China; ^2^ Department of Urologic Surgery, Chinese PLA General Hospital, The 309th Hospital of China People's Liberation Army, Beijing 100094, People's Republic of China

**Keywords:** RCC, p16^INK4A^, p14^ARF^, promoter methylation, clinical significance

## Abstract

The inactivation of *p16^INK4A^* and *p14^ARF^* via promoter methylation has been investigated in various cancers. However, the clinical effects of *p16^INK4A^* and *p14^ARF^* promoter methylation on renal cell carcinoma (RCC) remain to be clarified. The pooled data were calculated and summarized. Finally, an investigation of 14 eligible studies with 1231 RCC patients and 689 control patients was performed. Methylated *p16^INK4A^* and *p14^ARF^* were observed to be significantly higher in RCC than in control subjects without malignancies (OR = 2.77, P = 0.005; OR = 11.73, P < 0.001, respectively). Methylated *p16^INK4A^* was significantly associated with the risk of RCC in the tissue subgroup, but not in the serum and urine subgroups. Methylated *p16^INK4A^* was significantly associated with tumor size. We did not find that *p16^INK4A^* promoter methylation was associated with sex, tumor grade, lymph node status, and tumor histology. Methylated *p14^ARF^* was significantly correlated with sex and tumor histology. Three studies reported that *p16^INK4A^* methylation was not significantly correlated with the prognosis of RCC. The results suggested that *p16^INK4A^* and *p14^ARF^* promoter methylation may be correlated with the carcinogenesis of RCC, and that methylated *p14^ARF^*, especially, can be a major susceptibility gene. We also found the different clinicopathological significance of *16^INK4A^* and *p14^ARF^* in RCC. Additional studies with sufficient data are essential to further evaluate the clinical features and prognostic effect of *p16^INK4A^* and *p14^ARF^* promoter methylation in RCC.

## INTRODUCTION

Renal cell carcinoma (RCC) is one of the most common cancers of the human urinary system. Based on cancer statistics, approximately 62,700 new cases will be reported in clinics, with approximately 14,240 deaths in the USA in 2016 [1]. Clear cell renal cell carcinoma (ccRCC) is the most common histological type of RCC, accounting for 70% to 75% of all RCCs [2]. Most patients with RCC are symptom-free in the early stage, and more than 50% of RCCs are found coincidentally by physical examination and imaging [3]. Approximately 30% of the patients with RCC have developed metastases, and the average 5-year survival rate is just 12.3% [4].

Epigenetic and genetic changes are identified to be significantly associated with cancer [5, 6]. DNA methylation is an important mechanism of epigenetic alterations involved in gene expression, which is closely associated with the carcinogenesis and progression of various carcinomas [7–9]. The transcription repression of the gene via CpG island methylation of the promoter can lead to the downregulation of gene expression [10, 11]. Located at chromosome 9p21, cyclin-dependent kinase inhibitor 2A (*CDKN2A*) has two alternative splicings, encoding the cell cycle regulatory proteins *p16^INK4A^* and *p14^ARF^*, which have a key function in regulating the activities of the retinoblastoma (RB) and p53 genes, respectively [12, 13]. *p16^INK4A^* and *p14^ARF^* as tumor suppressor genes are involved in the regulation of cell division and apoptosis, and the maintenance of cellular homeostasis [14]. The inactivation of *p16^INK4A^* and *p14^ARF^* through promoter methylation has been reported in many cancers [15–17]. Promoter methylation of *p16^INK4A^* and *p14^ARF^* has been shown in different sample types of RCC, including blood, urine, and tissue samples [18–21].

Although some studies involving *p16^INK4A^* and *p14^ARF^* promoter methylation included patients with RCC, the studies published in this field have had small sample sizes. In addition, whether *p16^INK4A^* and *p14^ARF^* promoter methylation is associated with clinical characteristics of RCC remains to be determined. Therefore, in this study, we performed a systematic meta-analysis to further evaluate the clinical significance of *p16^INK4A^* and *p14^ARF^* promoter methylation in RCC.

## RESULTS

### Study characteristics

**Figure 1:**
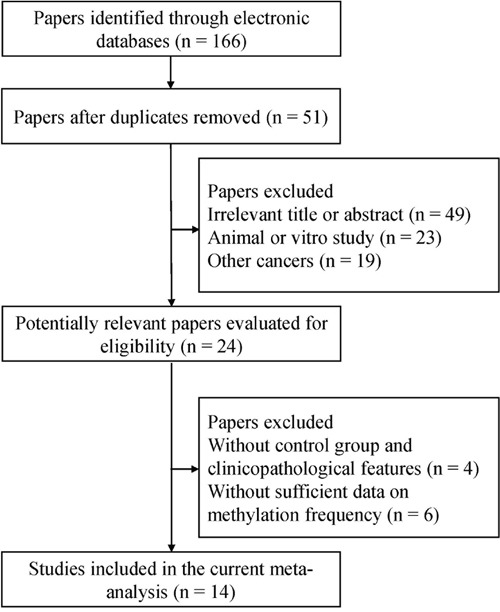
Flow chart of study selection

One hundred sixty-six potentially relevant studies were identified by the initial literature search. According to the inclusion criteria, a total of 14 studies involving 1231 RCC patients and 689 control patients [18–31] were included in the current analysis (Figure 1). Of these studies, which involved *p16^INK4A^* and *p14^ARF^* gene promoter methylation, nine studies evaluated the association between *p16^INK4A^* promoter methylation and RCC risk, five studies assessed the correlation between *p14^ARF^* promoter methylation and RCC risk, ten studies evaluated the relation between *p16^INK4A^* promoter methylation and clinicopathological features, and four studies evaluated the relation between *p14^ARF^* promoter methylation and clinicopathological features. The general characteristics of included studies are presented in Table 1.

**Table 1:** General characteristics of all eligible studies

First author	Country	Ethnicity	Method	Sample	Cancer		Control		OS	DFS	Gene
					M %	Total	M %	Total			
Kawada 2000 [30]	Japan	Asians	MSP	Tissue	2.2	91	-	-	-	-	*P14*
Esteller 2001 [31]	USA	Caucasians	MSP	Tissue	13.1	38	0	38	-	-	*P14*
Battagli 2003 [26]	USA	Caucasians	MSP	Tissue	18	50	0	27	-	-	*P14*
Battagli 2003 [26]	USA	Caucasians	MSP	Urine	18	50	0	12	-	-	*P14*
Dulaimi 2004 [25]	USA	Caucasians	MSP	Tissue	17	100	0	15	NS	-	*P14*
Hoque 2004 [21]	USA	Caucasians	QMSP	Urine	30.8	26	0	91	-	-	*P14*
Hoque 2004 [21]	USA	Caucasians	QMSP	Serum	5.55	18	3.33	30	-	-	*P14*
Hori 2007 [20]	Japan	Asians	MSP	Tissue	70.5	44	-	-	-	-	*P14*
Hauser 2013 [18]	Germany	Caucasians	*	Serum	14.3	35	0	54	-	-	*P14*
Kawada 2000 [30]	Japan	Asians	MSP	Tissue	3.3	91	-	-	-	-	*P16*
Romanenko 2002 [29]	Spain	Caucasians	MSP	Tissue	31.8	22	-	-	-	-	*P16*
Morris 2003 [27]	UK	Caucasians	MSP	Tissue	0	17	0	14	-	-	*P16*
Sanz-Casla 2003 [28]	Spain	Caucasians	PCR	Tissue	20	40	-	-	-	-	*P16*
Battagli 2003 [26]	USA	Caucasians	MSP	Tissue	10	50	0	27	-	-	*P16*
Battagli 2003 [26]	USA	Caucasians	MSP	Urine	8	50	0	12	-	-	*P16*
Dulaimi 2004 [25]	USA	Caucasians	MSP	Tissue	10	100	0	15	NS	-	*P16*
Hoque 2004 [21]	USA	Caucasians	QMSP	Urine	34.6	26	0	91	-	-	*P16*
Hoque 2004 [21]	USA	Caucasians	QMSP	Serum	22.2	18	0	30	-	-	*P16*
Arai 2006 [24]	Japan	Asians	MSP	Tissue	73.3	60	37	67	-	-	*P16*
Hori 2007 [20]	Japan	Asians	MSP	Tissue	6.8	44	-	-	-	-	*P16*
Vidaurreta 2008 [23]	Spain	Caucasians	MSP	Tissue	22.9	48	0	48	NS	NS	*P16*
Onay 2009 [19]	Turkey	Caucasians	MSP	Tissue	57.1	21	52.4	21	-	-	*P16*
Martino 2012 [22]	Austria	Caucasians	qPCR	Serum	46.5	157	44.2	43	-	NS	*P16*
Hauser 2013 [18]	Germany	Caucasians	*	Serum	25.7	35	16.7	54	-	-	*P16*

“*” Denotes detection method using methylation-sensitive real-time polymerase chain reaction; “-” indicates data not available; MSP: methylation specific polymerase chain reaction; PCR: polymerase chain reaction; qPCR: quantitative polymerase chain reaction; QMSP: quantitative methylation-specific polymerase chain reaction; M: methylation; OS: overall survival; DFS: disease-free survival; NS: not significant.

### Association between *p16^INK4A^* and *p14^ARF^* promoter methylation and RCC risk

**Figure 2:**
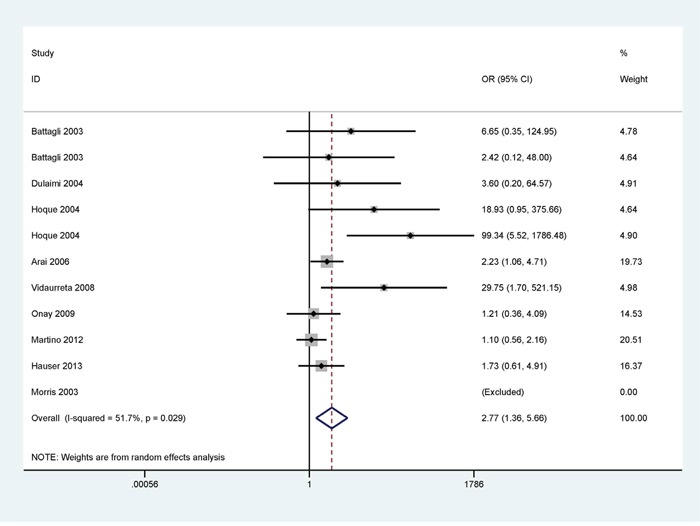
Forest plot showing the pooled OR from a random-effects model for *p16^INK4A^* promoter methylation in RCCs vs. nonmalignant controls

**Figure 3:**
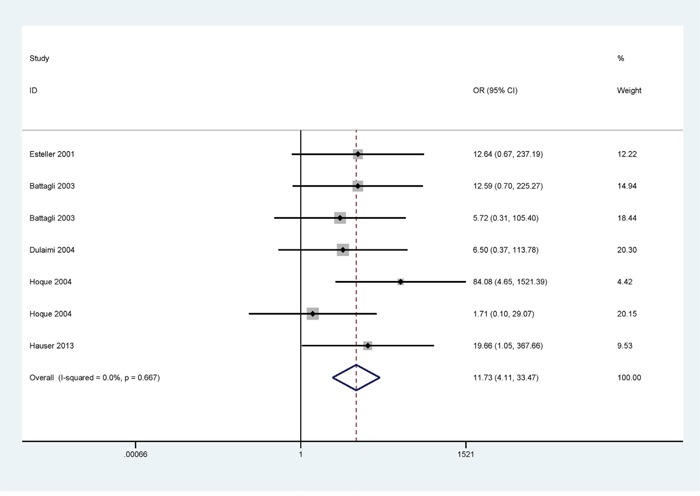
Forest plot showing the pooled OR from a fixed-effects model for *p14^ARF^* promoter methylation in RCCs vs. nonmalignant controls

When cancer patients were compared to control subjects, the result of *p16^INK4A^* promoter methylation with strong heterogeneity was conducted using a random-effects model (I^2^ = 51.7% and p = 0.029); under a fixed-effects model, no obvious heterogeneity was found for *p14^ARF^* promoter methylation (I^2^ = 0.0%; and p = 0.667) (Figures 2 and 3).

A significant association was found between *p16^INK4A^* promoter methylation and RCC (OR = 2.77, 95% CI = 1.36 - 5.66, P = 0.005), including in 582 of the cancer patients and 422 of the controls (Figure 2). The pooled OR of *p14^ARF^* promoter methylation in RCCs was significantly higher than in controls (OR = 11.73, 95% CI = 4.11 - 33.47, P < 0.001), including in 317 of the cancer patients and 267 of the controls (Figure 3).

### Subgroup analyses of *p16^INK4A^* promoter methylation in cancer patients versus control patients

According to sample type (tissue, serum, or urine), ethnicity (Caucasian or Asian), and testing method [methylation-specific polymerase chain reaction (MSP) and non-MSP], subgroup analyses were performed for *p16^INK4A^* promoter methylation with significant heterogeneity (Table 2).

**Table 2:** The pooled OR of *p16^INK4A^* and *p14^ARF^* promoter methylation and RCC

	Studies	Overall OR (95 CI %)	I^2^; P	P value	Cases	Controls	P (Egger test)
*p16INK4A*	9	2.77 (1.36 - 5.66)	51.7%; 0.029	0.005	582	422	0.011
*p14ARF*	5	11.73 (4.11 - 33.47)	0.0%; 0.667	< 0.001	317	267	0.193
Subgroup (*p16INK4A*)							
Ethnicity							
Asians	1	2.23 (1.06 - 4.71)	NA; NA	0.036	60	67	
Caucasians	8	3.40 (1.35 - 8.59)	57.6%; 0.016	0.009	522	355	
Sample							
Tissue	6	2.82 (1.61 - 4.95)	22.9%; 0.269	< 0.001	296	192	
Serum	3	1.66 (0.66 - 4.16)	45.1%; 0.162	0.28	210	127	
Urine	2	15.82 (0.41 - 608.03)	67.6%; 0.079	0.138	76	103	
Method							
MSP	5	2.06 (1.14 - 3.75)	0.0%; 0.815	0.017	298	156	
Non-MSP	4	5.85 (1.28 - 26.77)	77.2%; 0.002	0.023	284	266	

MSP: methylation-specific polymerase chain reaction; NA: not applicable; OR: odds ratio; 95% confidence interval (95% CI).

Subgroup analyses based on sample types showed that *p16^INK4A^* promoter methylation was significantly associated with RCC risk in tissue (OR = 2.82, 95% CI = 1.61-4.95, P < 0.001), but not in serum or urine (OR = 1.66, 95% CI = 0.66-4.16, P = 0.28; OR = 15.82, 95% CI = 0.41-608.03, P = 0.138, respectively). Subgroup analyses based on ethnicity and testing methods suggested that *p16^INK4A^* promoter methylation was significantly correlated with RCC risk in different ethnicities and by different testing methods (all P < 0.05).

### Meta regression and sensitivity analyses of *p16^INK4A^* promoter methylation in cancer patients versus control patients

Meta regression based on sample type (tissue, serum, or urine), ethnicity (Caucasian or Asian), and testing method (MSP or non-MSP) was performed to find the potential sources of heterogeneity (Table 3).

**Table 3:** Meta regression analysis of *p16^INK4A^* promoter methylation

Subgroup	Coefficient (95% CI)	t	P value
Testing method	2.835 (−0.681, 6.352)	1.97	0.096
Ethnicity	−0.225 (−2.909, 2.459)	−0.20	0.845
Sample material	−1.521 (−3.347, 0. 305)	−2.04	0.088

The results of meta-regression analysis showed that sample types, ethnicity, and testing methods did not explore the potential sources of heterogeneity (coefficient = −1.521, P = 0.088; coefficient = −0.225, P = 0.845; coefficient = 2.835, P = 0.096, respectively).

A sensitivity analysis was also conducted to evaluate the stability of the overall OR and the change of heterogeneity by deleting a single study. When a study from Hoque 2004 et al. ([21], urine) was removed, the pooled OR was not significantly changed (OR = 2.06, 95% CI = 1.41-3.02), with no obvious heterogeneity (I^2^ = 23.2%, and P = 0.237).

### Relation of *p16^INK4A^* and *p14^ARF^ promoter* methylation and clinicopathological features

We further determined whether *p16^INK4A^* and *p14^ARF^* promoter methylation status was associated with clinicopathological characteristics, such as sex, tumor grade, tumor stage, tumor size, lymph node status, and tumor histology. The fixed-effects model was used in relation to clinicopathological characteristics in cancer (all p > 0.1) (Table 4).

**Table 4:** The pooled OR of *p16^INK4A^* and *p14^ARF^* promoter methylation with clinicopathological features in RCC

Gene	Studies	Overall OR (95 CI %)	I^2^; P	P value	M (n)	RCCsmale	M (n)	RCCsfemale	P (Egger test)
*p14ARF*	3	0.48 (0.25 - 0.94)	29.1%; 0.238	0.032	41	168	25	76	0.769
*p16INK4A*	4	0.66 (0.31 - 1.38)	2.5%; 0.392	0.266	20	201	13	91	0.715
						Grade1-2		Grade 3-4	
*p14ARF*	3	2.13 (0.96 - 4.75)	17.5%; 0.297	0.063	40	111	15	72	0.613
*p16INK4A*	7	1.20 (0.58 - 2.45)	0.0%; 0.620	0.625	41	212	13	102	0.644
						Stage1-2		Stage 3-4	
*p14ARF*	1	1.03 (0.18 - 5.98)	NA; NA	0.97	6	35	2	12	NA
*p16INK4A*	4	1.00 (0.42 - 2.36)	0.0%; 0.786	0.999	25	104	11	52	0.211
						pT2-4		pT1	
*p14ARF*	4	0.92 (0.44 - 1.91)	7.4%; 0.356	0.815	16	94	41	180	0.229
*p16INK4A*	6	2.43 (1.10 - 5.35)	0.0%; 0.615	0.028	28	132	12	203	0.36
						Node+		Node-	
*p14ARF*	2	0.35 (0.04 - 2.83)	0.0%; 0.665	0.326	0	11	23	123	NA
*p16INK4A*	5	0.69 (0.18 - 2.69)	0.0%; 0.465	0.595	1	18	43	225	0.02
						CCRCC		Non-CCRCC	
*p14ARF*	4	0.38 (0.18 - 0.81)	0.0%; 0.607	0.012	34	185	25	100	0.294
*p16INK4A*	7	0.54 (0.29 - 1.00)	0.0%; 0.842	0.051	53	289	31	144	0.015

M: methylation; ccRCC: clear cell renal cell carcinoma; NA: not applicable; Node+: lymph node-positive status; Node-: lymph node-negative status; RCC: renal cancer carcinoma; pT: pathological T category of primary tumor; n: the number of samples; OR: odds ratio; 95% confidence interval (95% CI).

### Association of *p16^INK4A^* and *p14^ARF^* methylation and gender in cancer

The pooled OR from four studies suggested that *p16^INK4A^* promoter methylation was not significantly correlated with gender in RCC (OR = 0.66, 95% CI = 0.31-1.38, P = 0.266), including in 201 males and 91 females (Table 4). The pooled OR from three studies involving 168 males and 76 females suggested that *p14^ARF^* promoter methylation was significantly correlated with gender in RCC (OR = 0.48, 95% CI = 0.25-0.94, P = 0.032) (Table 4), indicating that it was lower in males than in females.

### Association of *p16^INK4A^* and *p14^ARF^* methylation and tumor grade in cancer

The pooled OR from seven studies and from three studies suggested that *p16^INK4A^* and *p14^ARF^* promoter methylation was not significantly correlated with tumor grade in RCC (OR = 1.20, 95% CI = 0.58-2.45, P = 0.625; OR = 2.13, 95% CI = 0.96-4.75, P = 0.063, respectively) (Table 4).

### Association of *p16^INK4A^* and *p14^ARF^* methylation and tumor stage in cancer

The pooled OR from four studies and from one study showed that *p16^INK4A^* and *p14^ARF^* promoter methylation was not significantly associated with tumor stage in RCC (OR = 1.00, 95% CI = 0.42-2.36, P = 0.999; OR = 1.03, 95% CI = 0.18-5.98, P = 0.97, respectively) (Table 4).

### Association of *p16^INK4A^* and *p14^ARF^* methylation and the pathological T category of primary tumor (pT) in cancer

The pooled OR from six studies including 132 pT2-4 patients and 203 pT1 patients suggested that *p16^INK4A^* promoter methylation was significantly correlated with tumor size in RCC (OR = 2.43, 95% CI = 1.10-5.35, P = 0.028) (Table 4), indicating that it was higher in pT2-4 than in pT1. The pooled OR from four studies suggested that *p14^ARF^* promoter methylation was not significantly correlated with tumor size in RCC (OR = 0.92, 95% CI = 0.44-1.91, P = 0.815), including 94 pT2-4 patients and 180 pT1 patients (Table 4).

### Association of *p16^INK4A^* and *p14^ARF^* methylation and lymph node status in cancer

The pooled OR from five studies and two studies showed that *p16^INK4A^* and *p14^ARF^* promoter methylation was not significantly associated with lymph node status in RCC (OR = 0.69, 95% CI = 0.18-2.69, P = 0.595; OR = 0.35, 95% CI = 0.04-2.83, P = 0.326, respectively) (Table 4).

### Association of *p16^INK4A^* and *p14^ARF^* methylation and tumor histology in cancer

The pooled OR from seven studies comprising 289 ccRCC and 144 non-ccRCC patients suggested that *p16^INK4A^* promoter methylation was not significantly associated with tumor histology in RCC (OR = 0.54, 95% CI = 0.29-1.00, P = 0.051) (Table 4). The pooled OR from four studies involving 185 ccRCC and 100 non-ccRCC patients demonstrated that *p14^ARF^* promoter methylation was significantly correlated with tumor histology in RCC (OR = 0.38, 95% CI = 0.18-0.81, P = 0.012) (Table 4), suggesting that it was lower in ccRCC than in non-ccRCC.

### Prognostic value of *p16^INK4A^* and *p14^ARF^* gene promoter methylation in RCC

The detailed overall survival (OS), and disease-free survival (DFS) data on *p16^INK4A^* or *p14^ARF^* gene promoter methylation as a prognostic factor for RCC were insufficient. The mean follow-up time for the participants ranged from 28 months [22] to 76 months [23] in this meta-analysis. Dulaimi et al. 2004 [25], Vidaurreta et al. 2008 [23], and Martino et al. 2012 [22] reported that *p16^INK4A^* methylation was not significantly associated with the prognosis in DFS or OS (Table 1). Dulaimi et al. 2004 [25] reported that *p14^ARF^* methylation was not significantly associated with the prognosis in OS (Table 1). More studies with sufficient data are necessary to further evaluate the prognostic value of *p16^INK4A^* and *p14^ARF^* promoter methylation in RCC.

### Publication bias

The Egger test was used to evaluate potential publication bias. The Egger test showed low publication bias for *p16^INK4A^* promoter methylation in cancer patients versus control patients, and in cancer in relation to lymph node status and tumor histology (P = 0.011, P = 0.02, P = 0.015, respectively) (Tables 2 and 4).

## DISCUSSION

The *p16^INK4A^* is formed from an alternative transcript of exons 1α, 2, and 3, whereas *p14^ARF^* is translated from alternative reading frames (ARF) consisting of exons 1β, 2, and 3 [32]. The silencing of *p16^INK4A^* and *p14^ARF^* can result in uncontrollable cell proliferation and tumor growth [32, 33]. Methylated *p16^INK4A^* and *p14^ARF^* have been investigated in various cancers, including RCC [18], esophageal squamous cell carcinoma [34], melanoma [35, 36], and gliomas [37]. Although numerous studies have been conducted to evaluate the role of *p16^INK4A^* and *p14^ARF^* promoter methylation in RCC, the results are still inconsistent and controversial. Kasahara et al. [38] found that the frequency of the methylated *p14^ARF^* was 0% in RCC. Hori et al. [20] found that the frequency of the methylated *p14^ARF^* was 70.5% in RCC. Morris et al. [27] reported that the frequency of *p16^INK4A^* promoter methylation was 0% in RCC. Arai et al. [24] reported that the frequency of *p16^INK4A^* promoter methylation was 73.3% in RCC. Therefore, we conducted this study of all available articles to further evaluate the effects of *p16^INK4A^* and *p14^ARF^* promoter methylation in RCC.

Analysis of the pooled OR showed that *p16^INK4A^* and *p14^ARF^* promoter methylation were significantly higher in patients with RCC than in control subjects, suggesting that *p16^INK4A^* and *p14^ARF^* inactivation via promoter methylation may play an important role in the tumorigenesis of RCC. Interestingly, *p14^ARF^* promoter methylation had a higher OR value (OR = 11.73) than that of *p16^INK4A^* promoter methylation (OR = 2.77) in cancer patients versus control patients, suggesting that RCC can be more susceptible to *p14^ARF^* promoter methylation.

When RCCs were compared to nonmalignant samples, the heterogeneity of *p16^INK4A^* promoter methylation was high (I^2^ = 51.7%, P = 0.029). According to sample type (tissue, serum, or urine), ethnicity (Caucasian or Asian), and testing method (MSP and non-MSP), subgroup analyses and meta-regression were used to explore the possible sources of heterogeneity. Analysis showed that subgroup analyses and meta-regression failed to find heterogeneity. Moreover, based on subgroup analyses of sample types, a significant association was observed between *p16^INK4A^* promoter methylation and tissue subgroup, but not in the serum and urine subgroups. The results should be carefully considered as only one study or two studies with a small number of samples involved in subgroup analyses. A sensitivity analysis was also performed in our study; when we deleted a study (Hoque 2004 et al., urine) [21], the overall OR was not significantly changed, with no significant heterogeneity, suggesting that our result was stable and reliable.

We further determined whether *p16^INK4A^* and *p14^ARF^* promoter methylation were correlated with clinicopathological features. Methylated *p14^ARF^* was significantly associated with gender, in which it was lower in males than in females, suggesting that female RCC patients can be more susceptible to *p14^ARF^* promoter methylation, whereas methylated *p16^INK4A^* had a similar frequency in males and females. Methylated *p16^INK4A^* was significantly associated with tumor size, in which it was higher in pT2-4 patients than in pT1 patients, suggesting that *p16^INK4A^* promoter methylation may play a key role in the pathogenesis of T2-4, whereas methylated *p14^ARF^* was not significantly correlated with tumor size. Methylated *p14^ARF^* was significantly associated with tumor histology, and it was lower in ccRCC than in non-ccRCC, suggesting that *p14^ARF^* promoter methylation had a decreased risk of ccRCC; whereas methylated *p16^INK4A^* had a similar frequency in ccRCC and Non-ccRCC. In addition, our findings showed that *p16^INK4A^* and *p14^ARF^* promoter methylation were not significantly associated with tumor grade, tumor stage, and lymph node status.

The prognostic data involving the pooled hazard ratio (HR) were insufficient and not available, as only three studies reporting showed that *p16^INK4A^* and *p14^ARF^* gene promoter methylation were not significantly correlated with the prognosis of RCC in OS or DFS [22, 23, 25]. More studies with sufficient data need to be done in the future.

The current study had several potential limitations. First, analysis of *p16^INK4A^* promoter methylation showed a slight publication bias in cancer versus control, and in cancer in relation to lymph node status and tumor histology. The articles with positive results are more often published than articles with negative results. The study was restricted to literatures published in English, which can lead to bias. In addition, because fluid samples from serum, plasma, and urine were limited, additional studies will be essential to evaluate the value of fluid detection in the future. Finally, the primary ethnic groups were Asian and Caucasian; thus, further studies using a larger variety of ethnic groups are warranted.

In conclusion, our study showed that RCC had a higher *p16^INK4A^* and *p14^ARF^* gene promoter methylation than did nonmalignant control patients. RCC had a higher *p16^INK4A^* promoter methylation in pT2-4 than in pT1. However, RCC had a lower *p14^ARF^* promoter methylation in males than in females, and was also lower in ccRCC than in non-ccRCC. Further large-scale studies with well-designed research are necessary to validate the role of *p16^INK4A^* and *p14^ARF^* promoter methylation in the prognosis and clinical effects of RCC patients in the future.

## MATERIALS AND METHODS

### Literature search

This meta-analysis was conducted based on the preferred reporting items for systematic reviews and meta-analyses (PRISMA) statement criteria [39] (Supplementary Table 1). We systematically searched for the relevant literature in the PubMed, EMBASE, EBSCO, and Cochrane Library databases without language restrictions. We used the following free text and their combinations: (kidney OR renal) AND (cancer OR tumor OR neoplasm OR carcinoma) AND (CDKN2A OR MTS1 OR P16 OR INK4A OR P14 OR ARF) AND (methylation OR epigene*) up to September 20, 2016. Finally, only full-text papers published in English were included in this study.

### Inclusion criteria

Eligible studies were selected in this meta-analysis if they met the following criteria: 1) patients were diagnosed with primary RCC; 2) although tissue specimens used must include surgically resected primary tumor samples, other samples, such as serum, plasm and urine, were used; 3) *CDKN2A* methylation included *p16^INK4A^* and *p14^ARF^* promoter methylation; 4) studies with sufficient data on *p16^INK4A^* and *p14^ARF^* promoter methylation frequency were selected to assess the association between *p16^INK4A^* and *p14^ARF^* promoter methylation and RCC; 5) to avoid duplicated publications, only the most recent paper or the most complete paper was included in the current study.

### Data extraction

We collected information from each eligible report regarding first author's name, country, ethnicity, testing method, sample type, methylation frequency, the number of samples, gender, tumor grade, clinical staging, pT, lymph node status, tumor histology, OS, and DFS. The whole data extraction was conducted independently by two authors, and minor disparities were solved by discussion.

### Data analyses

All statistical analyses were performed using STATA software (version 12.0, Stata Corporation, College Station, TX, USA). The pooled OR and 95 % confidence interval were calculated to assess the strength of the association between *p16^INK4A^* and *p14^ARF^* genes promoter methylation and RCC. Heterogeneity among studies was examined by Cochran test and the I^2^ test [40]. If I^2^ greater than 50% or p value less than 0.1 was considered as a measure of significant heterogeneity, then the random-effects model was applied in this study; otherwise, the fixed-effects model was used [41, 42]. The meta-regression and subgroup analyses were conducted to explore the source of heterogeneity. A sensitivity analysis was also performed to assess the contributions of an individual study on the overall OR by omitting one study [43]. Any possible publication bias was detected using the Egger linear regression test [44].

## SUPPLEMENTARY TABLE




