# Supplementary material for: Clinical significance of p16INK4A and p14ARF promoter methylation in renal cell carcinoma: a meta-analysis
Source: Oncotarget. 2017 Jun 28;8(38):64385–94. doi: 10.18632/oncotarget.18826 (PMC5610010; doi:10.18632/oncotarget.18826)
Supplement: Supplementary file 1 [file oncotarget-08-64385-s001.pdf]

Clinical significance of *p16*^{INK4A} and *p14*^{ARF} promoter methylation in renal cell carcinoma: A meta-analysis

SUPPLEMENTARY TABLE

Supplementary Table 1: PRISMA 2009 checklist

See Supplememtary File 1
